# Comparison of Titanium versus Polycel as Partial Ossicular Replacement Prosthesis: A Randomized Clinical Trial

**DOI:** 10.22038/ijorl.2021.52321.2775

**Published:** 2021-05

**Authors:** Mohammad Faramarzi, Masih Tale, Sheida Khosravaniardakani, Sareh Roosta, Ali Faramarzi

**Affiliations:** 1 *Department of Otorhinolaryngology-Head and Neck Surgery, Shiraz University of Medical sciences, Shiraz, Iran.*; 2 *Otolaryngology Research Center, Shiraz University of Medical Sciences, Shiraz, Iran.*; 3 *Student Research Committee, Shiraz University of Medical Sciences, Shiraz, Iran.*

**Keywords:** Hearing outcome, Ossiculoplasty, Partial ossicular replacement prosthesis, Polycel, Titanium

## Abstract

**Introduction:**

Each type of prosthesis for ossiculoplasty has its advantages and disadvantages, and the choice of the best material has been a matter of various studies. The present study aimed to make a comparison between the hearing outcomes of partial ossicular replacement prosthesis (PORP) using titanium versus Polycel prosthesis.

**Material and Methods:**

A total of 106 patients undergoing PORP as a second stage ossiculoplasty were analyzed in this study. Following that, they were randomly assigned to two groups of titanium (n=54) and Polycel (n=52) prosthesis. Subsequently, pre-and post-operative audiometric data were assessed based on the aim of the study.

**Results:**

In general, the post-operative air-bone gap within 20 dB was given to 63.5% and 55.6% of all ears in the Polycel and titanium groups, respectively, indicating a non-significant difference (P=0.407). Finally, no SNHL was observed in the groups.

**Conclusion:**

Overall, the hearing outcomes and the success rate of PORP are comparable between titanium and Polycel prostheses. Therefore, the selection of these prostheses could be based on the surgeons’ preferences, availability, and cost.

## Introduction

The disruption of the ossicular chain leads to conductive hearing loss, which could be secondary to various etiologies, including suppurative chronic otitis media, cholesteatoma, malignancies, trauma, and congenital disorders (1,2) . 

The surgical intervention for reconstructing the ossicular chain is referred to “ossiculoplasty”, which is currently considered among the most common otology procedures worldwide with favorable audiometric results (3,4).

 Ossiculoplasty could be performed to reconstruct ossicular chain, which are referred to as total ossicular replacement prosthesis. On the other hand, partial ossicular replacement prosthesis (PORP) is used when stapes is present (5,6). It is a fact that the endangered lenticular process of the incus is at risk from chronic otitis media. 

When the stapes is normal, the physical presence of normal body size and bulk of incus is a prerequisite for incus interposition. In this situation, incus interposition is the priority for ossiculoplasty in our center; accordingly, the sculptured incus connects the stapes into the malleus (7). However, the PORP is indicated in the complete absence of incus or too fragile remnant of the incus. Although the surgical procedure for PORP is nearly uniform among all centers and surgeons, the type of the applied prosthesis varies largely (8-11). 

Each type of prosthesis has its advantages and disadvantages, and therefore, the selection of the best material is a matter of importance in different studies. However, the issue remains controversial and requires further evaluation. 

The titanium prosthesis has been introduced to the era of ossiculoplasty in the 1990s and has been extensively used and studied with suitable and audiometric results (12,13).

Titanium prostheses have low weight and low impedance, making them appropriate for ossicular chain reconstruction. In addition, their application is feasible, and they have an appropriate biocompatibility profile, along with magnetic resonance compatibility (12,13).

Polycel is another prosthesis, which is made of thermal-fused polyethylene, and its advantages include biocompatibility and low immune-mediated response, feasibility, and easy surgical application in addition to inexpensive and appropriate hearing results (14). Although several studies have reported the audiometric results of ossiculoplasty using the titanium (5, 9,12-14) and Polycel^® ^(14,16,17), there is a scarce of proper and randomized clinical trials regarding the comparison of these two materials (8,18). 

Furthermore, the superiority of these two prostheses over each other has not been appropriately compared in the literature. Therefore, this study aimed at comparing the audiometric results of PORP between the titanium and Polycel prostheses in a series of patients with conductive hearing loss.

## Materials and Methods

1. Study Population 

This double-blind, randomized, and clinical trial was conducted from April 2016 to August 2019 in Dastgheib Hospital, which is a tertiary healthcare and referral center for ear, nose, and throat in southern Iran and is affiliated to Shiraz University of Medical Sciences, Shiraz, Iran. Initially, a total number of 140 ears were considered, which belonged to 140 patients who underwent PORP due to ossicular chain disturbance. The inclusion criterion was adults who underwent a second-stage PORP as ossiculoplasty. 

These patients required a second-stage surgery after primary tympanoplasty due to largely ossicular chain disturbance complete absence of incus. On the other hand, the patients with inadequate follow-ups in six months, pathologies (cholesteatoma), granulation tissue, severe fixation of stapes and footplate due to large tympanosclerotic plaques requiring stapedectomy in the second-look operation, and the presence of tympanic membrane perforation were excluded from the study. 

The study protocol was approved by the Medical Ethics Committee and Institutional Review Board of Shiraz University of Medical Sciences, Shiraz, Iran. 

Furthermore, this study was registered on the Iranian Registry for Clinical Trials website (www.irct.ir; IRCT2015082815496N19), and informed written consent forms were obtained from all patients and their guardians before participation in the present study. 

2.2 Randomization, Blinding, and Intervention 

In general, 140 patients were randomly assigned to two groups using a blocked randomization technique based on the type of prosthesis. Considering a block size of two, two possible methods, existed for assigning participants to BA or AB blocks. The A and B represented the titanium Kurz (TTP™-Vario system, Kurz GmbH, Dußlingen, Germany) and the Polycel (Sheehy Plastipore Polycel, Medtronic Xomed Inc., USA), respectively. Following that, 70 numbers (equivalent to the sample size in every group) were created within 0-9 using Excel software (Microsoft, Redmond, Washington). Moreover, AB and BA were selected for even (0, 2, . . ., 8) and odd (1, 3, . . ., 9) numbers, respectively. Ultimately, 54 and 52 ears were analyzed in the titanium and Polycel groups, respectively. In addition to patients, the assessors (i.e., the statistician and audiologist) were blind to the type of the utilized material.

2.3 Surgical Procedure 

All procedures were performed by the senior author. As routine in our center, the ossicular chain reconstruction was conducted in the second stage. The first stage included the middle ear cleaning from possible pathologies and tympanic membrane repair. The primary surgery for chronic otitis media can be carried out using three common surgeries, including canal wall-up mastoidectomy (CWU), tympanoplasty, and canal wall-down mastoidectomy (CWD). The operation was indicated based on the extent and type of pathologies, such as middle ear tympanosclerotic plaques, cholesteatoma, and the granulation tissue. Additionally, the second stage included ossicular reconstruction surgery through PORP, and the standard protocol of was applied to perform the surgery (6).

2.4 Outcome Measures 

All post-operative visits were performed by the same surgeon using microscopic otoscopic examinations. 

Audiometric data were analyzed one week before and six months after the surgery. In addition, pure tone audiometry and speech reception threshold (SRT) in 0.5, 1, and 4 kHz were checked to assess the hearing outcomes. It should be noted that the frequency of 3 kHz is not normally assessed in our center. Accordingly, it was determined as the mean of 2 and 4 kHz frequencies. Furthermore, several parameters were examined, including bone conduction (BC), pre- and post-operative air conduction (AC), and air-bone gap (ABG). In this study, successful hearing results were defined as the achievement of post-operative ABG of 20 dB or less (ABG closure ≤20 dB). Therefore, an ABG closure of <20 dB (i.e., hearing gain) within the range of 21-30 dB and >30 dB was considered successful, satisfactory, and unsuccessful, respectively (12). The frequency and hearing result of sensorineural hearing loss (SNHL) were also assessed in this study, and the SNHL was recognized as the post-ossicular chain reconstruction BC threshold, which was >10 dB poorer, compared to the pre-operative SNHL.

2.5 Statistical Analysis 

In this study, a minimum of 29 ears was assumed necessary in each study group according to a study conducted by Coffey et al., and 85% power to detect at least 7.2 dB differences between experimental and control groups regarding the post-operative ABG with an α equal to 0.05 (19). To increase the power of the study and recompense for non-evaluable patients, 70 ears were considered in each study group. The obtained data were analyzed in SPSS software (version 22.0, Inc. Chicago, Illinois, USA) and presented as mean±SD or proportions as proper. Furthermore, continuous variables with a normal distribution were compared utilizing the independent t-test between groups while those without a normal distribution were compared using Mann-Whitney U-test. Moreover, a comparison was made between continuous variables with a normal distribution within each group using the paired t-test. Additionally, the Wilcoxon signed-rank test was used for variables without a normal distribution, followed by comparing the proportions by the Chi-square test. A p-value less than 0.05 was considered statistically significant. 

## Results

**Fig 1:**
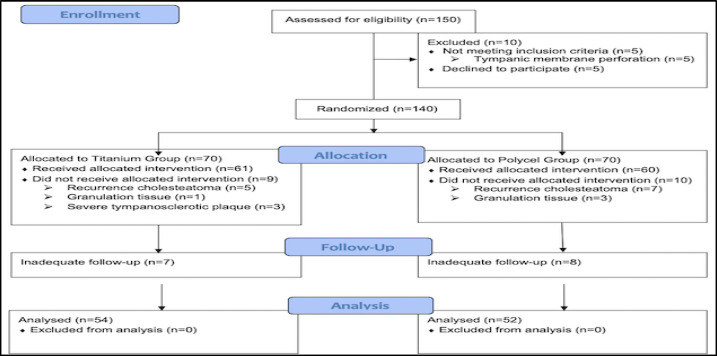
CONSORT trial flow diagram

Overall, 150 patients were evaluated for eligibility, and 10 cases were excluded from the study due to lack of satisfying the inclusion criteria (n=5) and unwillingness to contribute to the study (n=5). Accordingly, 140 patients, each with one affected ear, were randomly allocated to two study groups (70 ears per group). An 8-17- month (mean=14) duration was considered between the first and second operation, and 15 patients had inadequate follow-ups. Ultimately, 106 patients were included in the analysis (Fig.1).

According to Table 1, the patient's baseline features, such as age, gender, and the type of the primary operation were comparable between the two study groups (P>0.05).

**Table 1:** Characteristics of the patients

	**Titanium Group** **(n=54)**	**Polycel Group** **(n=52)**	**P-value**
Age (years)	35.7±13.5^a^	31.4±11.7	0.083
Gender			0.122
Male	18 (33.3)^b^	25 (48.1)	
Female	36 (66.7)	27 (51.9)	
Type of Primary Operation			0.958
Tympanoplasty	27 (50)	28 (53.8)	
CWU	22 (40.7)	20 (38.5)	
CWD	5 (9.3)	4 (7.7)	

Table 2 summarizes the hearing results; accordingly, the AC improved significantly in both groups (P<0.0001). Similarly, the BC (P<0.05), ABG (P<0.0001), and SRT (P<0.0001) represented considerable improvements after the operation. On the other hand, the baseline AC, ABG, and SRT were insignificant between the two groups (P>0.05). The results further demonstrated that improvements in AC, ABG, and SRT were comparable in both groups (P>0.05). Although the Polycel group had significantly lower baseline BC (P<0.001), no significant difference was observed in the two groups considering the BC gain after the operation (P=0.123). 

**Table 2:** Preoperative and postoperative hearing results

	**Titanium Group** (n=54)	**Polycel Group** (n=52)	**P-value**
AC^e ^(dB)			
Preoperative	48.4±11.4^b,c^	44.0±13.1^ c^	0.068
Postoperative	31.2±11.1	28.1±7.7	0.099
Gain	17.2±14.4	15.9±14.2	0.641
BC^e ^(dB)			
Preoperative	15.6±8.2^d^	10.0±5.7^ d^	0.0001
Postoperative	11.5±8.4	7.9±5.0	0.009
Gain	4.1±7.4	2.1±5.7	0.123
ABG^e ^(dB)			
Preoperative	32.7±9.9 ^c^	34.0±11.1^ c^	0.526
Postoperative	19.7±6.0	20.2±6.8	0.689
Improvement	13.1±10.9	13.8±12.2	0.756
SRT (%)			
Preoperative	47.9±10.3^ c^	45.6±13.2^ c^	0.319
Postoperative	28.7±10.8	25.9±8.6	0.144
Improvement	19.2±14.3	19.7±13.3	0.853

^a^N(%); ^b^Mean±SD; ^C^p<0.0001 and ^d^P<0.05 for within group comparison;^ e^Frequency of 0.5-3 kHz; AC**:** Air Conduction; BC**:** Bone Conduction; ABG**:** Air-Bone Gap; SRT**:** Speech Reception Threshold

The mean ABG improvement in different frequencies was compared between the two groups (Table 3); however, no significant difference was detected in this regard (P>0.05). As demonstrated in Table 4, the distribution of the ABG in the measured frequencies was not significant between the two groups (P>0.05). In addition, there was no significant difference between the titanium and Polycel groups in terms of the ABG success rate (55.6% vs. 63.5%, P=0.407). Eventually, no cases of SNHL were found in both groups. 

**Table 3:** Improvement of the air-bone gap in different frequencies

	**Titanium Group** (n=54)	**Polycel Group** (n=52)	**P-value**
Mean ABG Improvement (dB)			
0.25 kHz	15.7±14.2^a^	17.1±14.6	0.618
0.5 kHz	15.4±14.4	16.0±13.2	0.824
1 kHz	13.4±14.6	15.4±14.5	0.481
2 kHz	11.7±11.8	12.9±13.7	0.630
3 kHz	11.3±11.1	12.2±13.1	0.703
4 kHz	11.1±14.1	11.4±16.2	0.919

^a^Mean±SD; ABG: Air-Bone Gap

**Table 4:** Distribution of postoperative air-bone gap in frequencies of 500-3000 Hz

	**Titanium Group** (n=54)	**Polycel Group** (n=52)	**P-value**
Postoperative ABG (dB)			0.723
≤10	18 (33.3)^a^	20 (38.5)	
11-20	12 (22.2)	13 (25.0)	
21-30	19 (35.2)	13 (25.0)	
>30	5 (9.3)	6 (11.5)	

## Discussion

Generally, the selection of the best prosthesis in PORP operations is still a matter of debate. However, in this randomized clinical trial, no significant difference was found in hearing outcomes when using either titanium or Polycel as PORP. 

The results represented the success rates of 55.6% and 63.5% in the titanium and Polycel groups, respectively, which is in line with the range of 35%-100% in several other studies (8,14,20-23). 

Furthermore, the success rates were reported as 60%, 52%, and 51.5% by O’Connell et al. (13), Leet al. (22), and Schember et al. (24) who used titanium PORP, respectively, as well as 51% by Moon et al. (14) who applied Polycel PORP, which is relatively similar to our success rate. The selection of prosthesis may rely on the surgeon’s preferences, availability, and cost. Given that titanium prostheses are more expensive, compared to Polycel ones, it is recommended that the latter be used in PORP surgery. It should be noted that the cost of a titanium prosthesis is at least four times more than that of Polycel prosthesis (70 vs. 300 USD) in our country. The type of the previous operation, such as tympanoplasty, CWU, and CWD is considered a factor that affects the hearing result. Clinical reports associated with this issue are controversial. Some studies reported that the overall results after tympanoplasty and CWU are significantly superior, compared to CWD (24-26). 

Brenholz et al. (16) indicated that ossiculoplasty was more difficult due to the severity of the primary disease in CWD and the absence of the posterior canal wall. Based on the findings of another study, no statistically significant difference was found in hearing results regarding the type of primary surgery (23), which was consistent with the result of the present study. In the present study, the success rate was the highest in the CWU, followed by tympanoplasty and CWD (61.9%, 58.2%, and 55.6%, respectively); however, the difference was not statistically significant (P=0.906). Regarding the strength of the current study, it is a randomized clinical trial. Furthermore, all surgeries were performed by an academic otology surgeon, which led to the omission of the surgical skill as a confounding factor. In addition, there was no difference in the experience level as a confounding factor. However, this study has some limitations, such as short-term follow-ups and a relatively low number of participants. Nevertheless, no significant difference was observed in the trend within the two groups.

## Conclusion

In conclusion, it was found that hearing outcomes might be the same when utilizing either titanium or Polycel prosthesis as PORP by the surgeons. Therefore, the choice of these prostheses could be based on the surgeon’s preferences, availability, and cost. Eventually, if a satisfactory post-operative hearing outcome can be achieved, it is unnecessary to seek the latest and most expensive type of prosthesis.
